# Deep Learning on MRI Images for Diagnosis of Lung Cancer Spinal Bone Metastasis

**DOI:** 10.1155/2021/5294379

**Published:** 2021-07-14

**Authors:** Xiaojie Fan, Xiaoyu Zhang, Zibo Zhang, Yifang Jiang

**Affiliations:** ^1^Department of Orthopedics, Fourth Hospital of Hebei Medical University, Shijiazhuang 050011, Hebei, China; ^2^Department of Respiratory, Fourth Hospital of Hebei Medical University, Shijiazhuang 050011, Hebei, China

## Abstract

This paper aimed to explore the adoption of deep learning algorithms in lung cancer spinal bone metastasis diagnosis. Comprehensive analysis was carried out with the aid of AdaBoost algorithm and Chan-Vese (CV) algorithm. 87 patients with lung cancer spinal bone metastasis were taken as research subjects, and comprehensive evaluation was made in terms of preliminary classification of images, segmentation results, Dice index, and Jaccard coefficient. After the case of misjudgment on whether there was hot spot was excluded, the initial classification accuracy of the AdaBoost algorithm can reach 96.55%. True positive rate (TPR) was 2.3%, and false negative rate (FNR) was 1.15%. 45 MRI images with hot spots were utilized as test set to detect the segmentation accuracy of CV, maximum between-cluster variance method (OTSU), and region growing algorithm. The results showed that the Dice index and Jaccard coefficient of the CV algorithm were 0.8591 and 0.8002, respectively, which were considerably superior to OTSU (0.6125 and 0.5541) and region growing algorithm (0.7293 and 0.6598). In summary, the AdaBoost algorithm was adopted for image preliminary classification, and CV algorithm for image segmentation was ideal for the diagnosis of lung cancer spinal bone metastasis and it was worthy of clinical promotion.

## 1. Introduction

At present, cancer has become the primary cause of human death, and the incidence and mortality of lung cancer rank first among all cancers [1]. The occurrence of lung cancer is not easy to find, and about 50% of patients are already at the advanced stage (stage IV) at the time of diagnosis [2]. In recent years, with the advancement of science and technology, the five-year survival rate of patients with advanced lung cancer has gradually increased. Still, with the survival benefit of patients, the probability of bone metastasis and skeletal related events also increases [3, 4]. The emergence of bone metastasis indicates a shortened survival period and a serious decline in the quality of life of patients. In the treatment of lung cancer, the prevention and treatment of bone metastasis are also particularly important [5].

In lung cancer patients, the probability of bone metastasis is about 10%∼15%. After bone metastasis occurs, the average survival time of patients is only 6–10 months. Even after treatment, the 1-year survival rate of patients only accounts for 40% to 50% [6, 7]. 50% of lung cancer patients with bone metastases occur in the spine, and the rest often occur in the femur, ribs, and sternum. Bone metastasis of lung cancer is due to bone resorption caused by osteoclasts, usually manifested as osteolytic bone metastasis, which accounts for about 70% of malignant tumor bone metastases [8]. In clinical studies, only 50% of patients with bone metastases have clinical symptoms, usually accompanied by severe bone pain and skeletal related events (spinal cord compression, pathological fractures, hypercalcemia, etc.) [9]. Pathological fracture is the first symptom of patients with lung cancer bone metastasis, and hypercalcemia is a major cause of death of lung cancer bone metastasis.

The current diagnostic methods for lung cancer spinal bone metastasis include radionuclide imaging, X-ray, CT/enhanced CT, and MRI [10]. Radionuclide imaging technologies mainly include emission computed tomography technology (ECT) and positron emission tomography-computed tomography technology (PET-CT). ECT technology is highly sensitive in the detection of bone metastasis of lung cancer, and the missing rate is low. However, it is also prone to false positives in the detection of other bone lesions, and it is less specific in the diagnosis of bone metastasis. PET-CT has high sensitivity and higher specificity than ECT, but it is expensive and poor in generality in clinical testing. The X-ray operation is simple, the price is low, and it has certain specificity in the detection of bone metastases, but the sensitivity is poor in the detection of some early bone metastases. It is difficult to find early metastasis, and it is easy to miss the diagnosis due to the concealment of the intramedullary cortex [11]. CT/enhanced CT is more sensitive than X-ray; it can display the bone destruction caused by bone metastasis more accurately and can better reflect the relationship between the diseased tissue and the surrounding blood vessels and nerves. However, it is less sensitive to the early metastasis of cortical bone and the infiltration of bone marrow [10]. MRI has high sensitivity and specificity in the early metastasis of lesions. Its multiplanar and multisequence imaging features accurately show the occupied location, scope, and invasion of surrounding tissues in bone metastasis. It is the preferred tool for detecting bone marrow infiltration [12]. However, in the clinical practice of bone metastasis, MRI technology still needs to be fully developed.

In medical image processing, it mainly relies on the doctor's manual segmentation and subjective judgment currently. In clinical operations, faced with a large number of medical images, affected by fatigue and film reading experience, doctors are likely to have different judgments on the images, leading to missed and misdiagnosed situations in subsequent clinical treatment. With the development of computer technology, the computer-aided diagnosis technology of medical image processing based on machine learning has played an important role in clinical practice. Its automatic diagnosis technology greatly reduces the pressure of doctors to read the film, provides doctors with systematic observation information, and assists doctors in making correct decisions based on medical images [13]. Deep learning algorithms do not need to rely on clinicians to manually extract features. In clinical segmentation, only the MRI image of lung cancer needs to be input into the algorithm training to achieve automatic segmentation, detection, and recognition of the lesion. It contains two concepts: hierarchical structure and feature extraction. Moreover, it can quickly extract a large amount of information in medical images, and mine deep-level features of images [14], which play an increasingly important role in current clinical diagnosis and treatment. To realize the automatic diagnosis of patients with lung cancer spinal bone metastases, AdaBoost algorithm and CV algorithm were employed to perform preliminary classification and lesion segmentation on MRI images of patients. This study aimed to provide evidence for the early imaging diagnosis of lung cancer spinal bone metastases.

## 2. Materials and Methods

### 2.1. Research Subjects

87 patients with lung cancer spinal bone metastasis who were diagnosed at hospital from May 2016 to September 2019 and whose primary tumors were identified through pathology or clinical follow-up were selected as the research subjects. MRI was performed on each patient. Among the 87 patients, 51 were males, and 36 were females, aged 29–76 years, with an average age of 54 ± 11.3 years. The study had been approved by the Medical Ethics Committee of the Hospital. The patients and their families were aware of the study and had signed informed consent forms.

Inclusion criteria: I, patients older than 18 years old; II, patients suspected of having spinal tumors before MRI examination; III, patients who had not undergone surgery, radiotherapy, chemotherapy, or needle biopsy; IV, patients with lung cancer spinal bone metastasis confirmed by pathological biopsy after MRI; V, patients with complete clinical data.

Exclusion criteria: I, patients who had undergone surgery, radiotherapy, chemotherapy, or needle biopsy; II, patients who cannot undergo MRI scan or refuse to undergo MRI scan; III, patients with incomplete clinical data; IV, patients who were not suitable for this study for other reasons.

### 2.2. MRI Parameters

A 3.0T superconducting MRI scanner produced by Siemens, Germany, was utilized to scan and examine patients. The conventional fast gyro echo sequence (including sagittal T2-weighted imaging (T2WI) sequence, sagittal T2WI fat pressure sequence, and cross section) was adopted. After the lesions were found, a high-pressure syringe was utilized to inject Gadolinium diethylenetriamine penta-acetic acid at 2 mL/s at a dose of 0.1 mmol/kg. After the injection, an MRI scanner was employed to detect whether the drug absorption was abnormal. Siemens DCE-MRI setting parameters were as follows. TR = 450∼550 ms; TE = 120∼150 ms. The whole spine image was established. The scanned images were quantitatively analyzed to complete the measurement of the size, number, and brightness of the hot spots.

### 2.3. Segmentation Algorithm for Lung Cancer Spinal Bone Metastasis MRI Images

**Figure 1:**
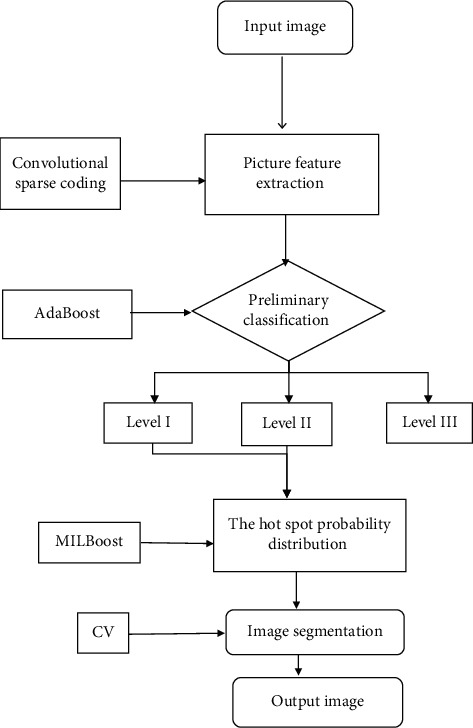
Flow chart of automatic segmentation algorithm.

In addition to manual segmentation, traditional bone metastasis scan-aided diagnosis generally adopts adaptive threshold method or region growth method to detect and segment hot spots [15]. However, in lung cancer spinal bone metastasis MRI images, the effect of bone marrow infiltration is usually manifested as low signal-to-noise ratio and convenient blur. It is difficult to obtain satisfactory segmentation results through traditional segmentation methods. To achieve automatic and accurate segmentation of hot spots on MRI images, firstly, convolutional sparse coding is combined to extract the depth features of the input MRI image. Then, the image classifier is trained through the AdaBoost algorithm to make preliminary judgments on the image (Figure 1). If the image is judged to be a normal image by the classifier, the algorithm stops, and no subsequent segmentation is performed. If it is judged to be a suspicious image, the hot spot classifier trained by the multi-instance algorithm is utilized to scan the suspicious image. The hot spot probability map of the original MRI image is generated according to the scan result. Finally, the hot spot probability map is binarized, which is taken as the initial contour of the image, and the level set method is employed to complete the precise image segmentation.

Traditional learning methods usually rely on manually extracting image features, which consumes manpower and time, and the judgment result is often affected by the personal experience of clinicians. As deep learning technology enters the field of computer vision, this type of problem has become simpler. Deep learning algorithms automatically extract image features in an unsupervised way and then simulate humans for image visual processing. By extracting image features layer by layer, the target image features can be expressed [16].

In this work, convolutional sparse coding is utilized to complete the feature extraction of lung cancer spinal bone metastasis MRI images. Convolutional sparse coding is a typical unsupervised learning algorithm in the field of deep convolutional networks. When an original image is input, convolutional neural coding can randomly extract a large number of small areas on the image. Assuming that the size of the extracted small areas is *l* × *l*, these areas will be an unlabeled vector {*x*_1_, *x*_2_,…, *x*_*n*−1_, *x*_*n*_}. Then, the problem of calculating the base of the input image with convolutional sparse coding can be transformed into the following optimization problem:

(1) $$\begin{array}{c} \underset{c,\phi }{\min}{\overset{n}{\underset{i=1}{\sum }}\left\|x_{i}-\overset{m}{\underset{j=1}{\sum }}c_{i}{,}_{j}\phi_{j}\right\|}_{2}^{2}+\lambda \overset{n}{\underset{i=1}{\sum }}\overset{m}{\underset{j=1}{\sum }}{\left\|c_{i}{,}_{j}\right\|}_{1}. \end{array}$$

In equation (1), *x*_*i*_ is the input vector, and *ϕ*_*j*_ is the base, and (*j* ∈ 1,2,…, *m* − 1, *m*). *c*_*i*_,_*j*_ is the excitation coefficient of the *x*_*i*_ to *ϕ*_*j*_. It is required to solve the minimum value of the objective function about *c* and *ϕ*, which can be divided into two parts for iterative and alternate calculation.

First, the base *ϕ* is fixed to find the minimum objective function about *c*. Then, the activation function *c* is fixed to find the minimum objective function about *ϕ*. The above two steps are looped until the function converges, and the following equation is adopted to extract the feature vector of the input image:

(2) $$\begin{array}{c} \underset{c}{\min}\overset{n}{\underset{i=1}{\sum }}{\left\|x_{i}-\overset{m}{\underset{j=1}{\sum }}c_{i}{,}_{j}\phi_{j}\right\|}_{2}^{2}+\lambda \overset{n}{\underset{i=1}{\sum }}\overset{m}{\underset{j=1}{\sum }}{\left\|c_{i}{,}_{j}\right\|}_{1}. \end{array}$$

After convolutional sparse coding is adopted to extract the depth features of the image, the AdaBoost algorithm is employed to make a preliminary judgment on the image. AdaBoost algorithm can well adjust the sample weight according to the weak classifier misclassification rate of the current sample distribution. A three-layer classification and regression tree (CART) is taken as the weak classifier of the AdaBoost algorithm.

In the preliminary judgment, if the output result of the AdaBoost algorithm sign (Result) > 0, it means that there are hot spots in the input image. If the output result sign (Result) < 0, it means that there is no hot spot in the image. Since the absolute value of the output result (and the degree of confidence) is different when the classifier is utilized for classification, if the degree of execution is too small, the classification result of the classifier is determined to be uncertain. Therefore, the bone scan results of the output image are divided into three categories: I) there must be a hot spot; II) it was uncertain whether there is a hot spot; III) there was no hot spot.

After AdaBoost algorithm is employed to classify and calculate the original MRI images, the images that do not have hot spots are excluded. Then, the multi-instance learning algorithm is adopted to scan the images. To improve the accuracy of the algorithm, semisupervised learning is employed for training. The specific operation is to mark out a small amount of rough data knowledge first and then conduct automatic exploration of fine knowledge through algorithms. In the multi-instance learning algorithm, the training set contains a positive bag and a negative bag. The positive bag requires at least one example to be a positive sample, and the negative bag requires all examples to be negative samples. The packet-level classifier can be represented by the following functions:

(3) $$\begin{array}{c} M\left(x_{j}\right)=\underset{l}{\max}\left[m\left(x_{jl}\right)\right]. \end{array}$$

In equation (3), *M*(*x*_*j*_) represents a classifier at the package level, *m*(*x*_*jl*_) represents a classifier at an example level, *j* represents the index of the package, and *l* represents the index of the example. The multi-instance algorithm is to learn an example-level classifier *m*(*x*_*jl*_). MILBoost algorithm is employed to optimize the loss function Loss(*m*) through the gradient descent method to train a large number of classifiers *m*_*t*_. The loss function Loss(*m*) it uses is in negative logarithmic form. It can be expressed by the following functions:

(4) $$\begin{array}{c} Loss\left(m\right)=-\overset{n}{\underset{j=1}{\sum }}\left[1\left(y_{j}=1\right)\log\;p_{j}+1\left(y_{j}=-1\right)\log\left(1-p_{j}\right)\right]. \end{array}$$

In equation (4), *p*_*j*_ represents the probability that the bag *j* is positive. Many *m*_*t*_ are combined into a strong classifier *m*(*x*_*jl*_), which can be expressed by the following function:

(5) $$\begin{array}{c} m\left(x_{jl}\right)=\sum_{j=1}^{n}\partial_{t}m_{t}\left(x_{jl}\right). \end{array}$$

In equation (5), the weight *m*_*t*_ represented by ∂_*t*_ is combined with the weight *m*(*x*_*jl*_) calculated by the function Loss(*m*), and the absolute value of the weight *m*_*jl*_ is employed to find the optimal weak classifier of the MILBoost algorithm. The weight ∂_*t*_ of the weak classifier is calculated again. After the calculation is complete, the final example-level classifier is acquired by adding the weak classifier and the previous strong classifier, which can be expressed by the following function:

(6) $$\begin{array}{c} m\left(x_{jl}\right)=\sum_{t=1}^{T}\partial_{t}m_{t}\left(x_{jl}\right). \end{array}$$

The above example-level classifier is adopted to perform a global scan on the lung cancer spinal bone metastasis MRI image, the scan result is taken as the distribution probability of the hot spot, and the hot spot probability map is used to realize the subsequent automatic segmentation of the hot spot.

The traditional bone metastasis scan-assisted diagnosis OTSU adaptive threshold segmentation method is susceptible to the interference of noise in the image, and it depends on the segmentation of each region in the early stage. The regional growth method needs to provide growth seed points and growth coefficients, and the segmentation results for different hot spots are not stable enough, and they are prone to leakage [17]. The level set method minimizes the energy function through iterative evolution and has good accuracy and topological characteristics in actual segmentation. According to the segmentation method, it can be divided into two categories, namely, the region-based segmentation method and the boundary-based segmentation method [18]. The boundary-based segmentation method uses the edge information of the image to attract the contour curve to expand the image boundary, but it is sensitive to the initial position of the contour and is easily affected by noise, and the segmentation effect is not very good [19]. The region-based segmentation method solves the above problems well. In this study, the CV model is employed to segment the MRI image of lung cancer spinal bone metastasis in the last step. The segmentation method can be represented by the following function:

(7) $$\begin{array}{c} \text{CV}\left(G\right)=\lambda_{a}{\int_{\Omega_{a}}\left|P\left(m\right)-g_{a}\right|}^{2}\text{dm}+\lambda_{b}{\int_{\Omega b}\left|P\left(m\right)-gb\right|}^{2}\text{dm}. \end{array}$$

In equation (7), Ω represents the image domain, and *G* represents a curve evolved on Ω. Ω_*a*_ and Ω_*b*_ represent the image area inside the curve and outside the curve. *P* represents a given gray image. *P*(*m*) represents the image gray value at the point *m*. *g*_*a*_ and *gb* represent means of the two areas. *λ*_*a*_ and *λ*_*b*_ represent two normal numbers.

After the hot spot distribution probability map is made by MILBoost algorithm, the hot spot probability map *P*(*m*) is binarized with *ρ* as the threshold. Then, the result is taken as the initial contour of the CV segmentation algorithm. The binarization method can be expressed by the following function:

(8) $$\begin{array}{c} \phi_{\text{initial}}=\phi \left(m,t=0\right)=\left\{\begin{array}{cc} g, & m\in \left\{m|P\left(m\right)<\rho \right\},\\ 0, & m\in \left\{m|P\left(m\right)=\rho \right\},\\ -g, & m\in \left\{m|P\left(m\right)>\rho \right\}. \end{array}\right. \end{array}$$

The value of *ϕ*_initial_ is set to 0.5, and the time step Δ*t* is set to 0.13. The CV function is evolved continuously through iterative calculation, and finally the segmentation of the initial image is complete. The relationship can be expressed by the following function:

(9) $$\begin{array}{c} \phi_{c+1}=\phi_{c}+\Delta t\frac{\partial \phi_{c}}{\partial t}. \end{array}$$

### 2.4. Criteria for Classification and Segmentation Results

First, two radiologists performed manual segmentation of MRI images of spine bone metastasis by double-blind method and compared the segmentation results after segmentation. If there was a difference, the result of the division shall be determined through a joint negotiation, and then the result of the division after the negotiation shall be the gold standard. Then, the preliminary classification results of the AdaBoost algorithm were compared with the gold standard. The accuracy, TPR, and FNR of the AdaBoost algorithm were analyzed. CV algorithm, OTSU algorithm, and region growing algorithm were employed to segment MRI images that were determined to contain hot spots. Jaccard coefficient and Dice index were adopted to judge the segmentation result. The calculation method can be expressed by the following functions:

(10) $$\begin{array}{c} J\left(M,N\right)=\frac{\left|M\cap N\right|}{\left|M\cup N\right|},\\ D\left(M,N\right)=\frac{2\left|M\cap N\right|}{\left|M\right|+\left|N\right|}. \end{array}$$

Here, *M* represented the area manually segmented by experts, that is, the real area, and *N* represented the area automatically segmented by different segmentation algorithms.

### 2.5. Statistical Analysis

SPSS17.0 version software was employed to analyze the image data of 87 patients with lung cancer spinal bone metastasis. The accuracy, TPR, and FNR of the initial classification of the CV algorithm were recorded, expressed as a percentage (%). SNK-q test was implemented, and *P* < 0.05 meant that the difference was statistically significant.

## 3. Results

### 3.1. Judgment on Classification Accuracy of AdaBoost Algorithm Preliminary

**Figure 2:**
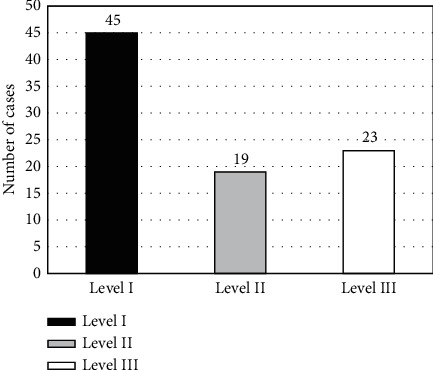
Preliminary classification results of the experts.

**Figure 3:**
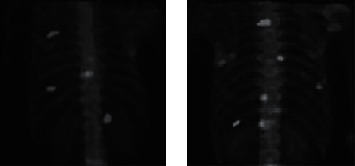
Preliminary classification level I images of AdaBoost algorithm. (It was certain that the image did contain hot spots.)

**Figure 4:**
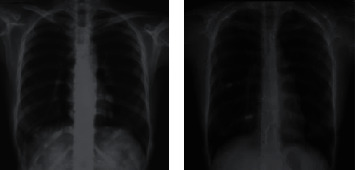
Preliminary classification level II images of AdaBoost algorithm. (It was not certain if the image contained hot spots.)

**Figure 5:**
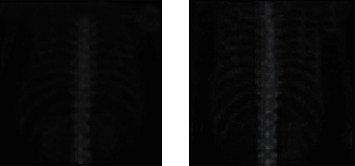
Preliminary classification level III images of AdaBoost algorithm. (It was certain that the image didn't contain hot spots.)

**Figure 6:**
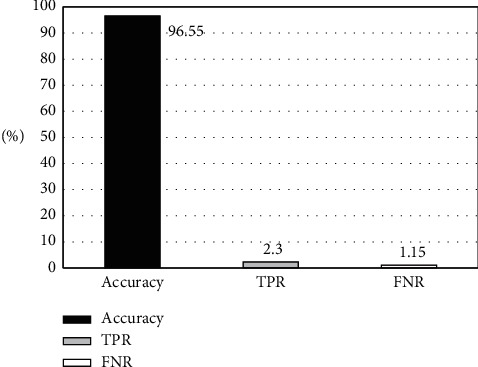
Preliminary classification results of AdaBoost algorithm.

87 lung cancer spinal bone metastasis MRI images were selected and firstly marked by two radiologists to be classified into 3 levels (Figure 2), and the results of the expert's marking were deemed as the gold standard (Figures 3–5). The results of the expert's annotation showed that level I contained 45 images: that is, there must be hot spots in 45 patients. Level II contained 19 images; that is, it was not sure whether there was a hot spot in the 19 patients. Level III contained 23 images; that is, 23 patients were determined to be without hot spot. The AdaBoost algorithm classification outcome of MRI images of patients with lung cancer spinal bone metastases was shown in Figures 3–5. The expert's gold standard was compared with the preliminary judgment classification result of the AdaBoost algorithm. The AdaBoost algorithm preliminarily judged that the accuracy of level I image classification was 89.4%, and the FNR was 3.5% (Figure 6). The accuracy of level II image classification was 87.9%, that of level III image classification was 92.1%, and the false positive rate was 2.9%. When the AdaBoost algorithm was employed for classification, since the classifier classified some of the level I and III images into level II images, the existence of image hot spots was uncertain, resulting in a decrease in overall accuracy. If the classifier did not take level I and level III images as level II images, the overall accuracy would be 96.55%.

### 3.2. Judgment on Segmentation Accuracy of Different Segmentation Algorithms

**Figure 7:**
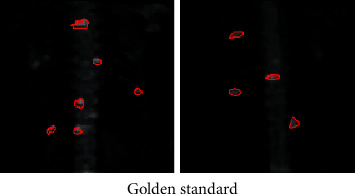
Expert manual segmentation results.

**Figure 8:**
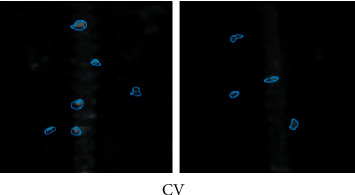
Segmentation results of CV algorithm.

**Figure 9:**
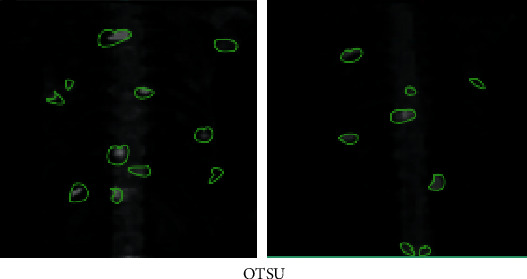
Segmentation results of OTSU algorithm.

**Figure 10:**
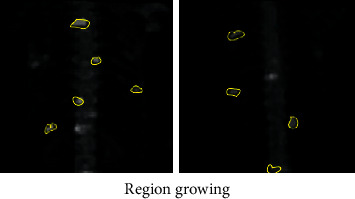
Segmentation results of region growing algorithm.

**Figure 11:**
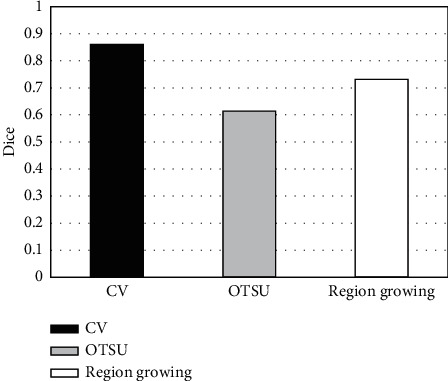
Dice index comparison of different segmentation algorithms.

**Figure 12:**
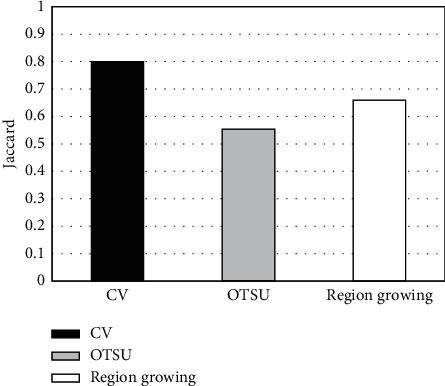
Comparison of Jaccard coefficients of different segmentation algorithms.

CV algorithm was compared with the traditional OTSU adaptive threshold segmentation method and region growing, and the segmentation accuracy of the three algorithms was compared. 45 level I images manually annotated by experts were taken as the test set (Figure 7), and experts were invited to annotate image hot spots. Then, three different segmentation algorithms were adopted to segment 45 MRI images, and the segmentation accuracy was measured according to Dice index and Jaccard coefficient (Figures 8–10). The results showed that the Dice index and Jaccard coefficient of the OTSU algorithm were 0.6125 and 0.5541, respectively. The Dice index and Jaccard coefficient of region growing algorithm were 0.7293 and 0.6598, respectively. The Dice index and Jaccard coefficient of the CV algorithm were 0.8591 and 0.8002, respectively. Dice index and Jaccard coefficient of the three segmentation algorithms were compared (Figures 11 and 12), and the differences were remarkable (*P* < 0.05).

## 4. Discussion

Artificial intelligence has been widely used in the medical field, and the application range of deep learning algorithms is extensive [20]. To study the adoption of deep learning algorithms in lung cancer spinal bone metastasis diagnosis, CV algorithm was employed to analyze the segmentation accuracy and diagnostic accuracy of lung cancer spinal bone metastasis MRI images. Moreover, OTSU adaptive threshold segmentation method and region growing method were introduced for comparison. 87 cases of lung cancer spinal bone metastasis patients whose primary tumors were identified through pathology or clinical follow-up were selected as the research subjects for MRI detection. First, the convolutional sparse coding was adopted to extract deep features from the patient's MRI image. Then, AdaBoost algorithm was applied to train the image classifier to make a preliminary judgment on the images. Finally, according to whether there were hot spots, the judgment results were classified into three levels, I, II, and III, which were compared with the gold standard manually marked by radiologists. It was found that the accuracy of the AdaBoost segmentation algorithm can reach 96.55% in the case of excluding level II, that is, the uncertainty of whether there was a hot spot as a misjudgment. After the preliminary judgment was made, the experts manually marked the 45 images classified as level I, which meant there must be hot spots, as the test set, to verify the segmentation accuracy of CV, OTSU, and region growing algorithms. Then, the Dice index and Jaccard coefficient were proposed for accuracy evaluation. The result showed that the Dice index and Jaccard coefficient of the CV algorithm were 0.8591 and 0.8002, respectively, which were evidently superior to OTSU algorithm and the region growing algorithm, which was similar to the study results of Zhang et al. Therefore, the CV algorithm was more accurate in segmenting lung cancer spinal bone metastasis MRI images.

## 5. Conclusion

AdaBoost algorithm was employed for preliminary classification of images, and the adopted CV algorithm for image segmentation had a favorable adoption prospect in the diagnosis of lung cancer spinal bone metastasis. This learning algorithm is worthy of clinical promotion. The results also provide information on the adoption of deep learning algorithms to the diagnosis of lung cancer spinal bone metastasis, as well as a theoretical basis for future studies. However, this work only analyzes and discusses the segmentation effect of the CV algorithm and does not conduct in-depth research on other algorithms. Moreover, the number of samples selected for the experiment is small, which cannot fully reflect the adoption prospects of the CV algorithm in lung cancer spinal bone metastasis diagnosis, and more in-depth research with more data is required in future work.

## Data Availability

No data were used to support this study.
